# Multi-frequency electrical impedance tomography and neuroimaging data in stroke patients

**DOI:** 10.1038/sdata.2018.112

**Published:** 2018-07-03

**Authors:** Nir Goren, James Avery, Thomas Dowrick, Eleanor Mackle, Anna Witkowska-Wrobel, David Werring, David Holder

**Affiliations:** 1 Medical Physics & Biomedical Engineering, University College London, London WC1E 6BT, UK; 2 Stroke Research Centre, Department of Brain repair and Rehabilitation, University College London Institute of Neurology, London WC1N 3BG, UK

**Keywords:** Brain imaging, Preclinical research, Stroke

## Abstract

Electrical Impedance Tomography (EIT) is a non-invasive imaging technique, which has the potential to expedite the differentiation of ischaemic or haemorrhagic stroke, decreasing the time to treatment. Whilst demonstrated in simulation, there are currently no suitable imaging or classification methods which can be successfully applied to human stroke data. Development of these complex methods is hindered by a lack of quality Multi-Frequency EIT (MFEIT) data. To address this, MFEIT data were collected from 23 stroke patients, and 10 healthy volunteers, as part of a clinical trial in collaboration with the Hyper Acute Stroke Unit (HASU) at University College London Hospital (UCLH). Data were collected at 17 frequencies between 5 Hz and 2 kHz, with 31 current injections, yielding 930 measurements at each frequency. This dataset is the most comprehensive of its kind and enables combined analysis of MFEIT, Electroencephalography (EEG) and Computed Tomography (CT) or Magnetic Resonance Imaging (MRI) data in stroke patients, which can form the basis of future research into stroke classification.

## Background & Summary

One of the most important challenges in stroke management and care is to differentiate haemorrhagic and ischaemic stroke to enable appropriate treatment^1^. Ischaemic stroke can be treated through the use of thrombolytic or (clot-dissolving) agents, but the benefits are limited to the first six hours, with better patient outcomes for earlier treatment inside this window^2,3^. Further, thrombolytics are deleterious, or even potentially fatal, to patients suffering from haemorrhagic stroke, so it is essential to differentiate between the two stroke types through neuroimaging. Recanalisation through mechanical thrombectomy has recently become the standard of care for patients with anterior circulation ischaemic stroke, and as with thrombolytics, the surgery should be performed within six hours of onset^4,5^. CT and CT angiography is necessary to determine cases suitable for thrombectomy, which places further importance in early differentiation of ischeamic and haemorrhagic stroke, to ensure patients are placed on the correct diagnostic pathway. It is estimated that delays in admission to stroke centres, and to obtaining a CT or MRI scan mean that treatment rates in eligible patients can be as low as 4% to 10% (ref. 6). Thus there is a clear clinical need for new methods which can rapidly distinguish haemorrhagic from ischaemic stroke, without waiting for hospital admission and a CT or MRI scan.

Electrical Impedance Tomography is a safe, portable and inexpensive imaging method, which can produce images of the internal conductivity of an object, by injecting insensible electrical currents through surface electrodes at frequencies between several kHz and several MHz. EIT has the potential to image brain function and pathology, with current applications including localisation of epileptic foci^7,8^, imaging fast neural activity^9^ and for monitoring cerebral oedema, ischaemia and intracranial haemorrhage^10–13^. A contrast in the electrical impedance of ‘healthy’ brain tissue, blood and ischaemic brain tissue (Ω_*blood*_ <Ω_*brain*_<Ω_*ischaemia*_) has been shown experimentally, and it is these spectral differences which EIT attempts to exploit^14^. EIT has the potential to provide an inexpensive portable unit for use in ambulances or GP surgeries which would revolutionise thrombolytic management of stroke by providing imaging at the point of contact.

The purpose of this dataset is to provide rich neuroscientific data to aid the development of EIT imaging methods in acute stroke. The clinical scenario dictates that data must be collected at a single time point in the absence of a reference or ‘pre-stroke’ image, which prevents the use of conventional “Time Difference” algorithms for dynamic imaging^15^. It is therefore necessary to use frequency difference EIT algorithms^16,17^, which use data collected at a single point in time and image changes across frequency. Whilst these methods have been demonstrated in simulation and in phantoms, they have not yet successfully translated to human studies, largely due to increased sensitivity to errors in modelling electrode shape and position, and systematic errors in impedance spectra of the tissues^18–21^. Development of methods robust to these errors is difficult without available quality MF-EIT data with representative experimental errors. To address this problem, a multi-frequency EIT dataset has been collected in 23 stroke patients and 10 healthy volunteers, in collaboration with the Hyper Acute Stroke Unit (HASU) at University College London Hospital (UCLH). Recordings were made in 10 patients presenting with ischaemia, eight with a haemorrhage and five with other classification, with the majority of measurements taken within 24 h of onset. Data were collected using the UCL ScouseTom system^22^ from 32 EEG electrodes at 17 frequencies between 5 Hz and 2 kHz with 930 individual impedance measurements at each frequency. It is possible to extract EEG signals from these recordings as it is recorded simultaneously. Radiological reports and corresponding CT and MRI scans are also included, in order to correlate the EIT data with the clinical diagnosis. Thus EEG inverse source modelling is possible through the combination of the EEG data and structural MRI and CT scans within the repository. As well as directing MFEIT reconstruction algorithms, this dataset also addresses the lack of a sufficient training dataset for the development of Electrical Impedance Spectroscopy (EIS) machine learning approaches.

## Methods

### Participants

All experiments were performed at the UCLH HASU and were approved by the University College London Research Ethics Committee, the London - Harrow Research Ethics Committee (REC favourable opinion reference number:15/LO/0092) and NHS/HSC R&D (IRAS ID: 168765). Patients were selected using the following criteria:

Inclusion criteria:

- Adult patient, at least 18 years of age, with no upper age limit
- Clinical diagnosis of cortical ischaemic stroke or lobar intracerebral haemorrhage
- The stroke has a greatest axial diameter >1.5 cm on CT/MRI, OR the patient has an National Institutes of Health Stroke Scale (NIHSS) score of five or greater
- Able to undergo the EIT assessment within 7 days of onset

Exclusion criteria:

- Expected to require critical transfer or intervention, Intensive Therapy Unit (ITU) admission or to be transferred out of the HASU for any other reasons
- Unlikely to tolerate the procedure (e.g. agitation on admission), or a very high likelihood of death within 48 h of stroke onset
- Additional medical illness (such as Epilepsy, skin problem, head or face metal implants) or technical aspect (such as no bed available on HASU) that interferes with EIT assessments

Potential participants were first identified by the clinical care team, who also initiated first discussions with the patient. If they were willing to participate, written consent was obtained. Where the patient lacked the capacity to give consent, assent was given by a member of the family. The inclusion criteria combined with the rate of consent/assent resulted in an approximate 20% participation rate. Experiments were also performed on ten healthy subjects after obtaining written consent.

### EIT Hardware

An EIT system typically comprises an electrode array, current source, voltage measurement unit and switching circuitry to direct the injection current to a particular subset of electrodes. The EIT system used in this study was developed at UCL^22^ and uses a Keithley 6221 Current Source (Keithley Instruments, Cleveland, Ohio, USA), BioSemi EEG Recorder (Biosemi, Amsterdam, The Netherlands), EasyCap EEG electrodes (EasyCap, Germany) and custom circuit boards for current routing and system control.

**Figure 1:**
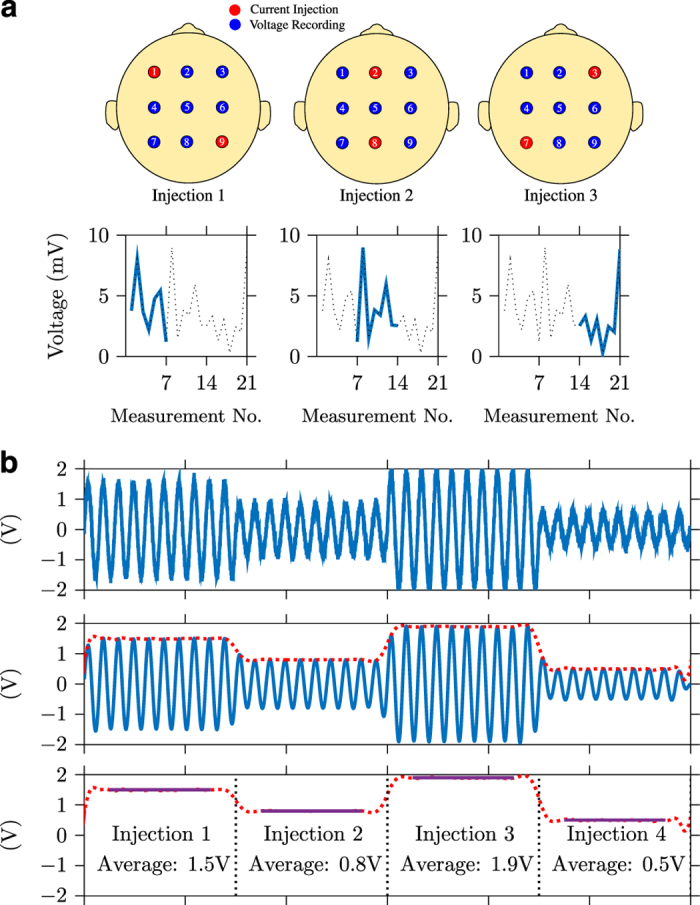
The EIT data collection and demodulation process. **a** Simplified EIT data collection with three current injection pairs out of nine total electrodes. For each injection pair, voltage recordings are made on the remaining seven electrodes, to produce a total of 21 measurements in this example. **b** Data processing pipeline on a single electrode in this example for four different injection pairs. Top: Raw data are imported from BioSemi bdf file, middle: filtering around the injection frequency and demodulation using the Hilbert transform (red line), bottom: segmentation into individual injections and averaging. The edges of the the waveform are excluded from averaging to remove switching and filtering artefacts.

A sinusoidal current, with an amplitude and frequency typically in the 100 *μ*A and kHz ranges, is injected between a pair of electrodes, referred to as an *injection*, and voltages are measured at all electrodes in parallel, Fig. 1a. A single EIT *measurement* is the demodulated voltage amplitude at a single electrode, averaged over a certain number of periods of the waveform, see Fig. 1b. The electrodes used for injection are typically excluded from the measurement set. A complete set of measurements, referred to as a *protocol*, is collected by repeating the current injection at a number of different injection pairs. The entire set of *n* voltage measurements, equal to the number of injection pairs multiplied by the number of measurement electrodes, is referred to as a *frame* of data.

### Electrode arrangement and application

**Figure 2:**
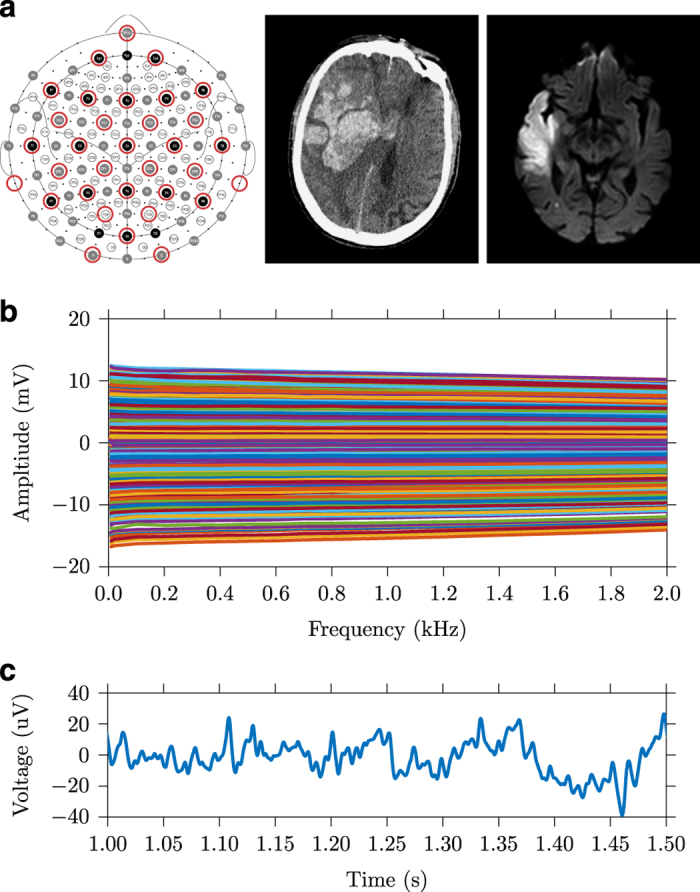
Example data for a single patient. (**a**) positions of the 32 electrodes based for EIT and EEG recordings on the extended 10-10 system, and example CT and MRI images, showing haemorrhage (P11) and ischaemia (P6) and respectively (**b**) Full spectrum EIT dataset with 930 voltages at 17 frequencies between 5 Hz and 2 kHz (**c**) A single channel of extracted EEG data.

Voltages were recorded on a total of 32 EEG electrodes (EasyCap, Germany) arranged in the configuration used in previous stroke EIT studies which includes 21 locations from the EEG 10–20 standard and 11 additional electrodes^23^. For these experiments, the locations were updated to match the nearest equivalents in either the 10-10 or 10-5 extensions^24^. The common electrode was placed on the forehead at position NFpz, above the nasion fiducial point, detailed in Fig. 2a. The Biosemi Active Two system implements a driven right leg circuit, which required an additional electrode, which was placed on the forehead 20 mm closer to the right temple at position NFp2. Nominal Cartesian coordinates for these positions are also given in *_electrodes.tsv* in Data Citations 1, Data Citations 2, Data Citations 3, Data Citations 4.

Prior to data collection, each electrode site was cleaned with surgical spirit, abraded using Nuprep gel (Weaver and Co., USA) and Elefix conductive paste (Nihon Kohden, Japan) was used to affix the electrodes. To ensure the quality of the electrode contact at each site, the contact impedance was estimated at 1 kHz and the electrode site was reabraded until all electrode sites met a 1–3 kΩ criteria used in previous EIT stroke studies^25^. This contact check was repeated prior to removal of the electrodes, to indicate any electrodes whose contact had altered during recording.

### EIT Data Collection

For this dataset, a total of 31 injections were chosen to maximise the overall magnitude of the voltages recorded, and the number of independent measurements^19^. This yields a total of 992 measurements, and a total of 930 carried through for further analysis after exclusion of measurements on injection channels.

Measurements were taken using seventeen frequencies between 5 Hz and 2 kHz, Table 1, with the current amplitude at each frequency set to the maximum allowed under the guidelines set out in IEC 60601-1 (ref. 26). In order to reduce the total data collection time, the number of current injection periods applied was reduced at lower frequencies. A full spectrum recording with all 930 voltage measurements collected at all 17 frequencies (for a total of 15,810) was collected for a total of three frames over the course of 20 minutes, and are stored with the *−MF* suffix. This frequency range spans both the operating bandwidth of the ScouseTom system and the region where the tissue conductivities demonstrate the largest differences^19,25^. A second reduced spectrum recording was collected at three frequencies, 200 Hz, 1.2 kHz and 2 kHz, for a total of 60 frames collected over 25 min, stored with the suffix *−TD*. This increased number of frames improves the accuracy of the measurements through an increased number of averages as well as giving a clearer picture of the noise present in the measurements.

### Repeated "Time Difference" Datasets

In some patients there was a higher probability that the pathology would evolve over time, such as Haemorrhagic transformations in large territory infarct after thrombolysis, or ICH which tend to develop secondary expansion. In these cases it was routine to perform a further CT or MRI scan within 24 h as part of the monitoring protocol at the HASU. In these instances the EIT recording session was also repeated as close to 24 h after the previous recording as was feasible, giving both a new full spectrum *−MF* and reduced spectrum *−TD* dataset. It is thus possible to correlate the changes in EIT data with the radiology reports. This second recording session was also repeated for every healthy volunteer, to characterise the variation arising from reapplication of the electrodes aside from any physiological changes. Datasets in patients with repeated recording sessions were tagged with additional *A* and *B* suffixes for the first and second recording respectively, *e.g. P12B* represents the second dataset recorded in patient 12.

### Data processing

The EEG data from the .bdf source files were converted to a double precision array, with dimensions *n*_*electrodes*_ x *n*_*samples*_. *n*_*electrodes*_ was 32 in all cases, *n*_*samples*_ was equal to the recording time in seconds multiplied by the sampling frequency. The digital triggers, also extracted from the .bdf file, were used to partition the data according to frequency and current injection pair as dictated by the injection protocol. This partitioning resulted in 31 segments for each different pair of injection electrodes, which were further subdivided into 17 subsections for each frequency. This whole pattern was repeated three times, producing three frames of data.

Next, the data within each segment were demodulated and averaged to provide a single modulus and phase value, shown in Fig. 1 for a single frequency on a single channel. First the signal was filtered with a zero-phase bandpass filter to remove noise and any DC offset. This signal was then demodulated using the Hilbert transform to provide the instantaneous amplitude and phase across each segment. Both ends of each segment were then trimmed of the number of samples equivalent to impulse response of the filter, to remove any artefacts arising from both the filtering and the switching of electrode pair. The remaining signal was then averaged to provide the final amplitude and phase measurement at each frequency on all 32 electrodes per current injection pair. The data were then reshaped into the matrices *BV* (magnitude values) and *PA* (phase values) with dimensions *n*_*protocol*_ x *n*_*frequencies*_ x *n*_*frames*_. The choice of filter was optimised based on the frequency and measurement time, to ensure a sufficient number of samples remained for averaging after trimming the edges of the data segments. A Butterworth filter with a bandwidth of 50 Hz either side of the carrier frequency was chosen, unless the impulse response was greater than 25% of the length of the data segment. In these cases an Finite Impulse Response (FIR) filter with a Blackman-Harris window was chosen with order equal to 25% of the data segment length. To prevent attenuation of the carrier frequency, at frequencies below 100 Hz, the bandpass filter was split into individual low and high pass filters, Table 1.

The gain of the BioSemi has a known frequency dependence, resulting from the anti-aliasing filter with the −3dB point at 3 kHz. To account for these changes across frequency, demodulated values were multiplied by an appropriate scaling factor^22^. Similarly, as the injected current amplitude is different across frequency, the results were normalised to maximum current amplitude at 2 kHz. The real component of the voltages were then extracted from the magnitude *BV* and phase *PA*, as only resistive changes were expected within the frequency range^19,25^. Finally, the values from all three collected frames were averaged together, and the final dataset is presented in mV. Data rejection was performed to remove measurements contaminated with artefacts arising from patient movement or faulty electrode contact. Notional reference values were calculated from ten datasets in healthy volunteers, and any subsequent values which differed by more than ±20 mV was removed. To maintain consistency of data dimensions, removed values were replaced with *NaN* values (“_NaN_” string in JSON format). A total of 171 measurements out of a possible 22,320, or 0.8%, were removed at this stage. The 930×17 (*n*_*protocol*_×*n*_*frequencies*_) matrix is the final output from all processing stages, Fig. 2b.

The *BV* and *PA* matrices output from the demodulation stage of the data processing were common to all experiments using the ScouseTom EIT system. Consequently the code for this section is located in the general *Load_Data* library http://github.com/EIT-team/Load_data. The normalisation and data rejection stages of the data processing are specific to these experiments and are found alongside the final processed data and other examples in the *UCLH_Stroke_EIT_Dataset* repository, which has been archived on Zenodo in Data Citation 1.

### EEG data

In each recording there is up to a minute of voltage recording before and after EIT injection in which EEG is the only signal present and is thus easily extracted. The signals are filtered using a second order Butterworth bandpass filter with 2 and 200 Hz cut off frequencies, and a 50 Hz notch filter, Fig. 2c. This is performed using the function *Extract_EEG* within the repository Data Citation 1. To a certain extent, recovery of the EEG signal *during* EIT injection is as simple as removing the EIT carrier frequency through low pass filtering. However, removal of the artefacts caused by switching of the current injection electrodes requires further processing such as template matching and removal^27^.

### MRI and CT data

As part of the clinical diagnostic procedure in the HASU, each patient underwent an MRI and/or CT scan (DICOM format). All data were anonymised by removing any identifying data from the DICOM file, using DICOM Cleaner. All relevant clinical data (former lesions, co-morbidities, etc.) were collected together with medical records, and concentrated in a Case Record Form (CRF) for future analysis in accordance with Good Clinical Practice requirements. Only data relevant for this study were extracted from these forms, and subsequently anonymised according to UK Data Service guidance. All reports and imaging data are stored in a Zenodo repository (Data Citation 5).

### Code availability

The hardware, firmware and software to control the ScouseTom EIT system is open-source and is found at https://github.com/EIT-Team/ScouseTom, or archived at http://doi.org/10.5281/zenodo.1009061, it is based on the Arduino platform and the PC software is written in MATLAB. A more detailed description of this system is presented in ref. 22. The data processing software to extract the demodulated voltage amplitude from the EEG system files is written in MATLAB and can be found in the repository http://github.com/EIT-team/Load_data or archived at http://doi.org/10.5281/zenodo.1009056. The BioSig library http://biosig.sourceforge.net/ is required for portions of this processing code for file access. All further processing steps, from demodulation to creation of the EITDATA data records, along with examples and detailed usage notes are found in the GitHub repository http://github.com/EIT-team/Stroke_EIT_Dataset, an archival copy of which has been deposited to Zenodo in Data Citation 1.

## Data Records

The folder structure is dictated by the GitHub repository archived in Data Citation 1, which contains all processing code, metadata and usage examples alongside the final processed data. As the size of the total dataset is over 150 GB, it was split into the following repositories: Patient data (Data citations 2, Data Citations 3), Subject data (Data citation 4) and Radiology data (Data citation 5). Only Data Citation 1 is required for use of the final processed EIT data.

### EIT Data

The final processed data for all patients and subjects is presented in a structure named *EITDATA* available in Data Citation 1, stored in both .mat and .json format with the name “*UCL_Stroke_EIT_Dataset*”. This is organised as a 1×34 structure with the following fields:

**NameTag**: Experiment or Patient identifier. Patients are labelled with the tag *P#* where # is the order in which the patients were consented for this study. In some instances clinical factors prevented data collection after consent, which resulted in a non continuous list shown in Table 2. Similarly the healthy subjects are tagged *S#* where # is the order of data collection.

**Classification**: The broad diagnosis category (Healthy, ischaemia, haemorrhage).

**SubClassification**: All distinct clinical categories (Healthy, small ischaemia, big ischaemia, small haemorrhage, big haemorrhage).

**VoltagesFull**: Real demodulated voltages (mV), averaged across all frames, in 930x17 array (*n*_*protocol*_×*n*_*frequencies*_).

**VoltagesCleaned**: VoltagesFull with *NaN* values replacing removed elements.

**RemovedChannels**: Information on which measurements were removed during data cleaning.

**Diagnosis**: Summary of diagnosis.

**StudyID**: Internal UCL reference, for correlating with other clinical data.

**Comments**: Additional comments regarding data collection or diagnosis.

Parameters common to all measurements which are necessary for both graphing and reconstructing EIT images are stored in a separate structure *EITSETTINGS* saved in the same .mat file with the following fields:

**Freq**: EIT carrier frequencies in Hz *n*_*frequencies*_×1

**Protocol**: EIT Measurement protocol *n*_*protocol*_×4, where each row represents an individual measurement. The columns represent the corresponding electrodes for this measurement [CS+ CS- V+ V-]. For example the first row is [1, 5, 32, 33], where current is injected between electrodes 5 and 32, with voltage measurements taken between electrodes 1 and 33.

**Electrode positions**: Nominal X, Y and Z coordinates of each measurement electrode, in the coordinate system of the corresponding segmentation.

### Raw Data Files

The full experimental data are organised into separate folders for each recording session by *NameTag* (Data citations 2, Data citations 3, Data citations 4). The raw data are saved in .bdf format, sampled at 16.384 kHz on all 32 channels simultaneously, over a dynamic range of +/− 262 mV with 24 bit resolution. For example, the full spectrum dataset for patient five has the file name *e.g. P5_MF1.bdf* for patient five, and the reduced spectrum dataset for patient 14 is stored is the file *P14_TD1.bdf*.

For both the *MF* and *TD* datasets, there are accompanying log files in both .txt or .mat format, which contain the EIT system settings necessary to demodulate the data. The data recorded for the contact impedance checks are stored in files with the *Z#* suffix, where # is the order of recording. This contact check was repeated a number of times before recording and once after recording. No log files were generated by the software for the contact impedance checks as the EIT system configuration is fixed.

### MRI/CT

MRI and CT scans are provided in NIfTI format (Data Citation 5) and are organised according to the Brain Imaging Data Structure format (BIDS) specification^28^, with the BIDS Subject number matching the patient number used elsewhere in this study. The exact imaging sequences and protocols used varied between patients dependent upon clinical demands.

## Technical Validation

Signal to noise ratio (SNR) is important factor in determining the performance of both conventional “Time difference” and Multi Frequency EIT methods. In simulation, MF images have been successfully reconstructed with an SNR of 30 dB^29^, and with noise equivalent to 44 to 48 dB^19^. However, these MFEIT algorithms are particularly sensitive to spectral errors, or systematic errors across frequency. Therefore the most important characteristic of the EIT system is that the SNR and amplitude is frequency invariant and was therefore a focus of previous work^22^. The normalisation for the gain of the BioSemi EEG system during data processing was validated in resistor phantom measurements, where the deviation from the expected values was 0.07%. A reciprocity error of 0.42%, comparable to state-of-the-art EIT systems further demonstrated the accuracy of the ScouseTom^30,31^. Equally important is the frequency invariance of the demodulation. As the *Collection Time* and *Injection Periods* changed across frequency (Table 1), it was not possible to use the same filter design in each case. The filter selection was validated by demodulating a simulated signal matching the expected amplitude, DC offset, frequency, phase and time, and comparing the results with the true value. The maximum amplitude and phase error was 0.001% and 0.0017% respectively at 10 Hz, and less than 10^−5^% for 100 Hz and above. This simulation study is found in the testing folder of the http://github.com/EIT-team/Load_data repository.

The quality of the electrode contact was verified using two different methods before data collection and continuously monitored throughout recording. First the electrode “offset”, *i.e.* the voltage at DC on each channel, as displayed through the BioSemi software was maintained at a level below 50 mV. This was necessary both as a measure of the quality of electrode contact, and also to prevent clipping of the EIT voltages. Second, the contact impedance was estimated from the voltage on each electrode when used to inject current at 1 kHz, found in the *Z#* files in Data Citations 2, Data citations 3, Data citations 4, and the electrode application process was repeated until a value of 3 kΩ or less was obtained. To verify that there were no significant changes in contact impedance during recording, this estimate was repeated prior to removal of electrodes. For further validation the contact impedance was also estimated at each of the 17 carrier frequencies from the data in the *−MF* recordings.

**Figure 3:**
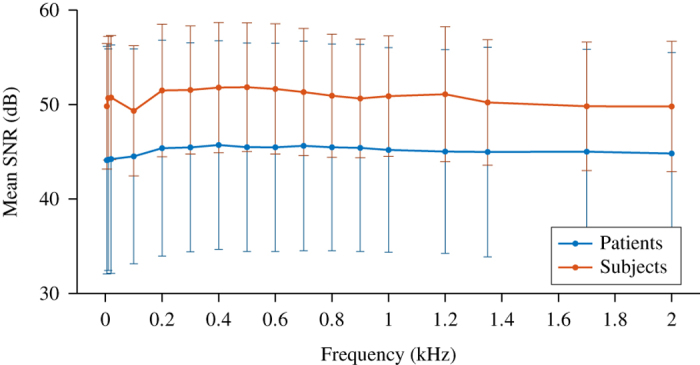
Signal to noise ratio across frequency (mean±standard deviation). There were no significant differences in SNR across frequency in both patient (N=18, n=24) and subject (N=10, n=20) recordings. There was an average 5 dB decrease in SNR in the patient recordings resulting from increased movement artefacts, although this was not significant at any frequency (*P*>=0.54).

The quality of the final data, Fig. 3, showed no significant changes of SNR across frequency for both subject and patient data, *P*=0.16 and *P*=0.52 (one way ANOVA) respectively. Increased artefacts from patient movement resulted in a mean 5 dB drop in SNR in patients compared to subjects, however this change was not significant at any frequency (*P*>=0.54). The <1% noise in these measurements was less than that applied in a successful simulation study^19^, less than half than that observed in the most directly comparable clinical recordings^25^, and within the range found using an order of magnitude greater current amplitude at 50 kHz^32^. Therefore this dataset meets the requirements for imaging both in terms of SNR and frequency invariance.

## Usage Notes

Detailed usage notes and example code is found in the readme for the GitHub repository archived in Data Citation 1, in which it is possible to fully replicate all processing stages to produce the final data set. To either extract the EEG data or to alter the EIT data processing it is necessary to download the files in Data Citation 2, Data Citation 3, Data Citation 4 and follow the folder structure described in Data Citation 1. So for example all files for Patient 10 are saved in *Patient_10.zip* in Data Citation 2 and should be extracted in the *Patients* folder, such that the raw *MF* full spectrum recording is at the following location: \Stroke_EIT_Dataset\Patients\Patient_10\P10_MF1.bdf.

The software described herein is based on the BioSig library http://biosig.sourceforge.net/, however the raw .bdf files could be loaded using other common EEG analysis software packages such as FieldTripToolbox http://www.fieldtriptoolbox.org/, EEGLAB http://sccn.ucsd.edu/eeglab/index.php or MNE https://www.martinos.org/mne/. Using any of these software packages it would be possible to extract the raw EEG for each recording without any knowledge specific to EIT. Re-referencing the voltages before analysis is recommended as a non clinical montage was used.

The processed EIT data for a single patient can be extracted in MATLAB as follows: plot(EITSETTINGS.Freq,EITDATA(7).VoltagesCleaned), which will produce the example data in Fig. 2a. This data coupled with the example head FEM in Data Citation 1 is sufficient to create EIT image reconstructions using existing linear alogrithms either in EIDORS, the most common EIT software^33^ or the other tools developed by the UCL group^34,35^. Further, these voltages are suitable for Multi Frequency EIT algorithms, thus enabling the first studies using these methods with clinical data^16,17^. It is the author’s hope that the data and examples found in this repository offer as low a barrier of entry as possible for researchers outside the brain EIT field.

## Additional information

**How to cite this article**: Goren, N. *et al*. Multi-frequency electrical impedance tomography and neuroimaging data in stroke patients. *Sci. Data* 5:180112 doi: 10.1038/sdata.2018.112 (2018).

**Publisher’s note**: Springer Nature remains neutral with regard to jurisdictional claims in published maps and institutional affiliations.

## Supplementary Material



## Figures and Tables

**Table 1:** Injected current parameters for each frequency used in the UCLH Stroke EIT Dataset, and filter settings used in subsequent data processing stages.

Frequency (Hz)	Amplitude (μA)	Periods per injection	Injection time (ms)	Filter(s)
5	45	32	6,400	LP IIR 7, HP IIR 2 LP IIR
10	45	32	3,200	LP IIR 7, HP IIR 2
20	45	32	1,600	LP IIR 7, HP IIR 2
100	45	32	320	BP FIR 2160
200	90	64	320	BP IIR 5
300	90	64	213	BP FIR 1470
400	90	64	160	BP FIR 1120
500	90	64	128	BP FIR 910
600	90	64	107	BP FIR 770
700	140	64	91	BP FIR 670
800	140	64	76	BP FIR 590
900	140	64	71	BP FIR 540
1,000	140	64	64	BP FIR 910
1,200	160	128	107	BP FIR 770
1,350	190	128	95	BP FIR 690
1,700	235	128	75	BP FIR 560
2,000	280	128	64	BP FIR 490
All 17 frequencies were used in the full spectrum *MF* recordings, and the highlighted frequencies were used in the reduced spectrum *TD* recordings.				

**Table 2:** EIT Data Summary.

Tag	Stroke to (EIT Interval)	Group	Scans Available (Time from Stroke)
P1	24 h	Small IS	CT (>12 h) MRI (24 h)
P3	27 h	Big ICH	CT (>12 h) MRI (24 h)
P4A	18 h	Big IS	CT (2 h) CT (26 h)
P4B	42 h		
P5	32 h	Small ICH	CT (2 h) MRI (>24 h)
P6A	36-48 h	Big ICH	CT (24 h) MRI (24 h)
P6B	25 days		
P9	2-3 days	Big IS	CT (>2 days) MRI (>3 days)
P11	15 h	Big ICH	CT (>12 h)
P12A	8 h	Small IS	CT (>3 h) CT (24 h)
P12B	24 h		
P15	3 days	Big IS	CT (>12 h) MRI (2 days)
P16	48 h	Small IS	CT (5 h) MRI (24 h)
P17	21 h	Small ICH	CT (>2 h) MRI (4 days)
P18	3 days	Small IS	CT (5 h) MRI (48 h)
P19A	15 h	Big IS	CT (>3 h) CT (20 h)
P19B	41 h		
P20	3 days	Small ICH	MRI (>2 days) CT (8 months prior)
P23A	>2 days	Big ICH	CT (>2 days) MRI (>3 days)
P23B	>6 days		
P24	27 h	Big ICH	CT (2 h) MRI (24 h)
P25A	12 h	Big IS	CT (1 hour) CT (25 h)
P25B	36 h		
P26	3-5 days	Big IS	CT (1-3 days)
S1B	N/A	Healthy	N/A
S2A	N/A	Healthy	N/A
S3B	N/A	Healthy	N/A
S4A	N/A	Healthy	N/A
S5A	N/A	Healthy	N/A
S6A	N/A	Healthy	N/A
S7A	N/A	Healthy	N/A
S8A	N/A	Healthy	N/A
S9A	N/A	Healthy	N/A
S10A	N/A	Healthy	N/A
Two data sets were collected from patients 4, 6, 12, 19, 23 and 25. IS=Ischaemia, ICH=Haemorrhage.			
